# Supplementary material for: The Systems Biology Research Tool: evolvable open-source software
Source: BMC Syst Biol. 2008 Jun 29;2:55. doi: 10.1186/1752-0509-2-55 (PMC2446383; doi:10.1186/1752-0509-2-55)
Supplement: Additional file 1 — SBRT Archive. An archive of the current version of the Systems Biology Research Tool. [file 1752-0509-2-55-S1.zip › sbrt-1.4.0/doc/developers_guide/api/sbrt/shell/text/math/class-use/LinearCombFormatV1.html]

Uses of Class sbrt.shell.text.math.LinearCombFormatV1


|  |  |  |
| --- | --- | --- |
| **Overview** \| **Package** \| **Class** \| **Use** \| **Tree** \| **Deprecated** \| **Index** \| **Help** | |  |
| PREV   NEXT | **FRAMES**    **NO FRAMES**     **All Classes** |  |


---


## **Uses of Class sbrt.shell.text.math.LinearCombFormatV1**

| Packages that use LinearCombFormatV1 | |
| --- | --- |
| **sbrt.shell.text.chem** | Provides classes and interfaces for defining formats for chemistry related objects. |
| **sbrt.shell.text.fba** | Provides classes and interfaces for defining formats for objects relevant to Flux Balance Analysis. |
| **sbrt.shell.text.math** | Provides classes and interfaces for defining formats for mathematical objects. |

Uses of LinearCombFormatV1 in sbrt.shell.text.chem

| Methods in sbrt.shell.text.chem that return LinearCombFormatV1 | |
| --- | --- |
| `LinearCombFormatV1<java.lang.String>` | `RevRxnFormatV1.getLinearCombFormat()`             Returns the format used for stoichiometric expressions. |
| `LinearCombFormatV1<java.lang.String>` | `IrrevRxnFormatV1.getLinearCombFormat()`             Returns the format used for stoichiometric expressions. |

Uses of LinearCombFormatV1 in sbrt.shell.text.fba

| Methods in sbrt.shell.text.fba that return LinearCombFormatV1 | |
| --- | --- |
| `LinearCombFormatV1<java.lang.String>` | `IrrevRxnFormatV1.getLinearCombFormat()`             Returns the format used for stoichiometric expressions. |
| `LinearCombFormatV1<java.lang.String>` | `RxnNameExprFormatV1.getLinerCombFormat()`             Returns the format used for linear combination. |

Uses of LinearCombFormatV1 in sbrt.shell.text.math

| Methods in sbrt.shell.text.math that return LinearCombFormatV1 | |
| --- | --- |
| `static LinearCombFormatV1<java.lang.String>` | `LinearCombFormatV1.getBasic()`             Returns a `LinearCombFormatV1` that does not format or parse the variables supplied to it. |

---


|  |  |  |
| --- | --- | --- |
| **Overview** \| **Package** \| **Class** \| **Use** \| **Tree** \| **Deprecated** \| **Index** \| **Help** | |  |
| PREV   NEXT | **FRAMES**    **NO FRAMES**     **All Classes** |  |


---
